# Abnormal blood microbiota profiles are associated with inflammation and immune restoration in HIV/AIDS individuals

**DOI:** 10.1128/msystems.00467-23

**Published:** 2023-09-12

**Authors:** Xiaoyan Guo, Zerui Wang, Mengmeng Qu, Yuntian Guo, Minrui Yu, Weiguo Hong, Chao Zhang, Xing Fan, Jinwen Song, Ruonan Xu, Jiyuan Zhang, Huihuang Huang, Enqiang Linghu, Fu-Sheng Wang, Lijun Sun, Yan-Mei Jiao

**Affiliations:** 1 Senior Department of Infectious Diseases, The Fifth Medical Center of Chinese PLA General Hospital, National Clinical Research Center for Infectious Diseases, Beijing, China; 2 Department of Gastroenterology, First Medical Center of Chinese PLA General Hospital, Beijing, China; 3 Center for Infectious Diseases, Beijing Youan Hospital, Capital Medical University, Beijing, China; Southern Medical University, Guangzhou, Guandong, China

**Keywords:** HIV, blood microbiota, inflammation, immune recovery, HIV reservoir

## Abstract

Although gut microbiota alteration and related blood microbe profiles in human immunodeficiency virus (HIV)-infected individuals are associated with the disease progression, how abnormal blood microbe profiles influence the inflammation and immune restoration are not fully understood. To address these issues, this study enrolled 24 healthy controls (HCs) and 91 HIV-infected individuals, including 30 treatment-naïve individuals (TNs), 31 immunological non-responders (INRs), and 30 immunological responders (IRs); subsequently, we analyzed blood microbe profiles using metagenomic sequencing and Olink proteomics technology, and identify inflammation-related proteins in peripheral blood samples of these individuals. The results showed increased translocation of microbes in the blood of TNs. This translocation did not return to normal level in either IRs or INRs who received antiretroviral therapy. In addition, *Porphyromonas gingivalis* significantly increased in TNs, IRs, and INRs compared to HCs. *P. gingivalis* was inversely associated with CD4^+^ T-cell counts, CD4/CD8 ratio, latency-associated peptide transforming growth factor-β1, and tumor necrosis factor-related activation-induced cytokine (TRANCE) and positively associated with HIV reservoirs. *Burkholderia multivorans* and *Bacillus thuringiensis* significantly decreased in TNs, IRs, and INRs compared to HCs and were positively associated with CD4^+^ T-cell counts and the CD4/CD8 ratio and negatively associated with HIV reservoir size and pro-inflammatory factors. We identified several species of microbes that were associated with CD4^+^ T-cell restoration on antiretroviral therapy, *Prevotella* sp*. CAG:5226, Eubacterium* sp*. CAG:251, Phascolarctobacterium succinatutens, Anaerobutyricum hallii, Prevotella* sp*. AM34-19LB,* and *Phocaeicola plebeius* were positively associated with HIV reservoir size and pro-inflammatory proteins. Another group of bacteria, *B. multivorans, B. thuringiensis, Vibrio vulnificus,* and *Acinetobacter baumannii*, which were negatively associated and pro-inflammatory proteins. In conclusion, different microbes within blood of HIV-infected individuals were found to be closely associated with persistent inflammation and immune restoration, suggesting the blood microbe profiles of HIV-infected individuals also influence disease progression.

**IMPORTANCE:**

The characteristics of blood microbiota in HIV-infected individuals and their relevance to disease progression are still unknown, despite alterations in gut microbiota diversity and composition in HIV-infected individuals. Here, we present evidence of increased blood microbiota diversity in HIV-infected individuals, which may result from gut microbiota translocation. Also, we identify a group of microbes, *Porphyromonas gingivalis*, *Prevotella* sp. *CAG:5226*, *Eubacterium* sp. *CAG:251*, *Phascolarctobacterium succinatutens*, *Anaerobutyricum hallii*, *Prevotella* sp. *AM34-19LB*, and *Phocaeicola plebeius*, which are linked to poor immunological recovery. This work provides a scientific foundation toward therapeutic strategies targeting blood microbiota for immune recovery of HIV infection.

## INTRODUCTION

Although antiretroviral therapy (ART) can effectively suppress viral replication and improve the immune status of the human immunodeficiency virus (HIV)-infected patients (1, 2), it is unable to fully solve the problems of residual immune activation and persistent inflammation caused by HIV infection (3) and is linked to several leading causes of morbidity and mortality of HIV infection (4). In fact, after successful ART, patients with HIV infection still have higher levels of interleukin (IL)-6, D-dimers, C-reactive protein, and soluble CD14 (sCD14), predictors of morbidity and mortality in patients on long-term ART (5 – 8). Persistent inflammation is also associated with increased cardiovascular events, accelerated liver disease, impaired immunological recovery, and mortality (5, 9, 10). However, the mechanism underlying HIV-related immune activation and inflammatory response is not fully understood.

Gut microbiota dysbiosis and microbial translocation are important causes of circulating inflammation in both treated and untreated individuals infected with HIV. Several studies have shown altered gut bacterial composition in HIV-infected individuals compared to healthy controls (HCs), with an increase in pro-inflammatory and potentially pathogenic bacteria and a decrease in beneficial bacteria (11 – 13). Particularly, these observations include an increased abundance of *Enterobacteriaceae*, which induce host inflammation upon infection and can utilize the by-products of this inflammation, namely reactive oxygen species from neutrophils and macrophages, to stimulate an increase in pro-inflammatory *Enterobacteriaceae*, which in turn exacerbates intestinal inflammation (14, 15). Studies have demonstrated that some species of *Prevotella*, which are abundant in individuals infected with HIV, enhance T helper (Th)17-mediated mucosal inflammation, have a pro-inflammatory effect, and are positively correlated with immune activation, while *Bacteroides*, which are reduced in these patients, can produce anti-inflammatory cytokines and have a protective effect against inflammation (12, 16).

Microbial translocation is another important cause of chronic inflammation. In particular, lipopolysaccharide, a component of the cell wall of gram-negative bacteria, is an indicator of microbial translocation and is significantly increased in individuals chronically infected with HIV and simian immunodeficiency virus-infected rhesus monkeys (17). Microbial translocation is also associated with increased activation of CD8^+^ T-cells and persistent failure of CD4^+^ T-cell reconstitution in patients receiving ART (18). In contrast, high inflammatory state of the intestinal mucosa and apoptosis of intestinal epithelial cells further promote intestinal microbial translocation (19).

However, most earlier studies have concentrated on how gut microbes affect inflammation and the course of the disease, ignoring the potential direct contribution of the blood microbiota to inflammation in individuals infected with HIV. A recent study found that the diversity of microbial fractions in the blood increases in the late stages of HIV infection, and ART ameliorates this blood microbiota perturbation (20). A longitudinal study revealed that the composition of translocated microbes in the blood affects the degree of recovery of CD4^+^ T-cells and the presence of persistent systemic inflammation (21). In addition, the characteristics of the blood microbiota at various disease stages of HIV infection and their relationship with inflammation have not been studied. It is unclear which specific microbial species in the blood are linked to HIV disease status and immune recovery, the potential connection between translocated microbiota and gut microbiota, whether blood microbes associated with disease progression are altered in the stool, and the extent of immune recovery after ART in persistent inflammation.

In the present study, we used shotgun metagenomic sequencing to elucidate the characteristics and functions of blood microbiota fractions in individuals infected with HIV at different disease stages and investigated their relationship with systemic inflammation and disease progression. In addition, gut microbiota composition in paired stool samples was investigated to determine whether the blood microbiota associated with disease progression is predominantly derived from gut microbial translocation.

## RESULTS

### Characteristics of the study population and study design

**Fig 1:**
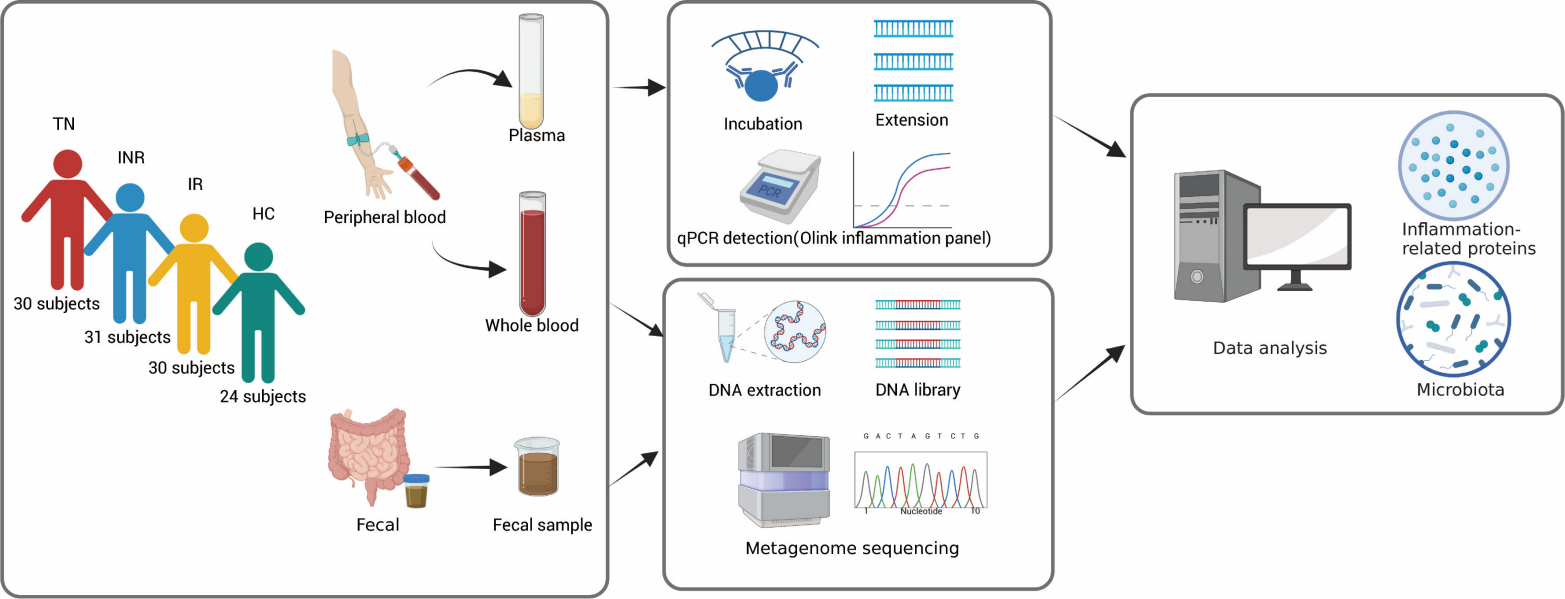
Study design. Peripheral blood and fecal samples were collected from TNs (*n* = 30), INRs ( *n* = 31), IRs (*n* = 30), and HCs (*n* = 24). The Olink inflammation panel was used to measure inflammation-related proteins in the plasma. Metagenome sequencing was used to detect the microbiota in the peripheral blood and feces. Data analysis of the microbiota and inflammation-related proteins was performed to explore their correlation and relevance to clinical parameters.

A total of 115 male subjects participated in the study, including 24 non-HIV-infected individuals as HCs. They were divided into four groups: treatment-naïve individuals (TNs), immunological non-responders (INRs), immunological responders (IRs), and HCs (Fig. 1). Peripheral blood and corresponding stool samples were collected simultaneously. The duration of ART was not significantly different between the IR and INR groups. No differences were observed in body mass index among the four groups (Table 1). The median CD4^+^ T-cell counts were 341, 317, 820, and 921 cells/µL in TNs, INRs, IRs, and HCs, respectively. Compared to IRs, INRs had significantly lower CD4^+^ T-cell and CD8^+^ T-cell counts, and CD4/CD8 ratio.

**TABLE 1:** Baseline characteristics of study subjects^*d*^ ^,*e*,^ ^*f*^

	TNs (*n* = 30)	INRs (*n* = 31)	IRs (*n* = 30)	HCs (*n* = 24)	*P* value
Age (years, IQR)	30 (26, 37)	44 (35, 51)	37 (30, 42)	32 (27, 43)	< 0.001^*a*^, 0.030^*b*^
Male gender (no.,%)	30 (100)	31 (100)	30 (100)	24 (100)	–
BMI (kg/m^2^, IQR)	22.62 (20.65, 25.95)	22.31 (21.45, 25.28)	22.47 (20.93, 25.93)	22.98 (21.52, 24.35)	0.937^*a*^
Viral load (log_10_ copies/mL, IQR)	4.20 (3.62, 4.91)	＜LDL	＜LDL	–	–
CD4^+^ T-cell count (cells/μL, IQR)	341 (183, 500)	317 (265, 332)	820 (637, 1,063)	921 (608, 1,156)	< 0.001^*a*^, < 0.001^*b*^
CD8^+^ T-cell count (cells/μL, IQR)	1,036 (718, 1,356)	787 (501, 991)	975 (705, 1,275)	790 (673, 1,039)	0.040^*a*^, 0.024^*b*^
CD4/CD8 ratio (IQR)	0.29 (0.21, 0.50)	0.38 (0.29, 0.48)	0.85 (0.67, 1.29)	1.14 (0.78, 1.50)	< 0.001^*a*^, <0.001^*b*^
Duration of ART (years, IQR)	–	7 (4, 8)	6 (5, 8)	–	0.931^*c*^

^*a*^ Kruskal-Wallis test.

^*b*^ Kruskal-Wallis test, INRs vs IRs.

^*c*^ Mann-Whitney *U* test.

^*d*^ Continuous variables are expressed as median (IQR), and categorical variables are expressed as the number of cases (%).

^*e*^ "–”, not applicable.

^*f*^ HCs, healthy controls; BMI, body mass index; LDL, lower than detectable level.

### HIV infection and ART affect the distribution and composition of blood microbiota

**Fig 2:**
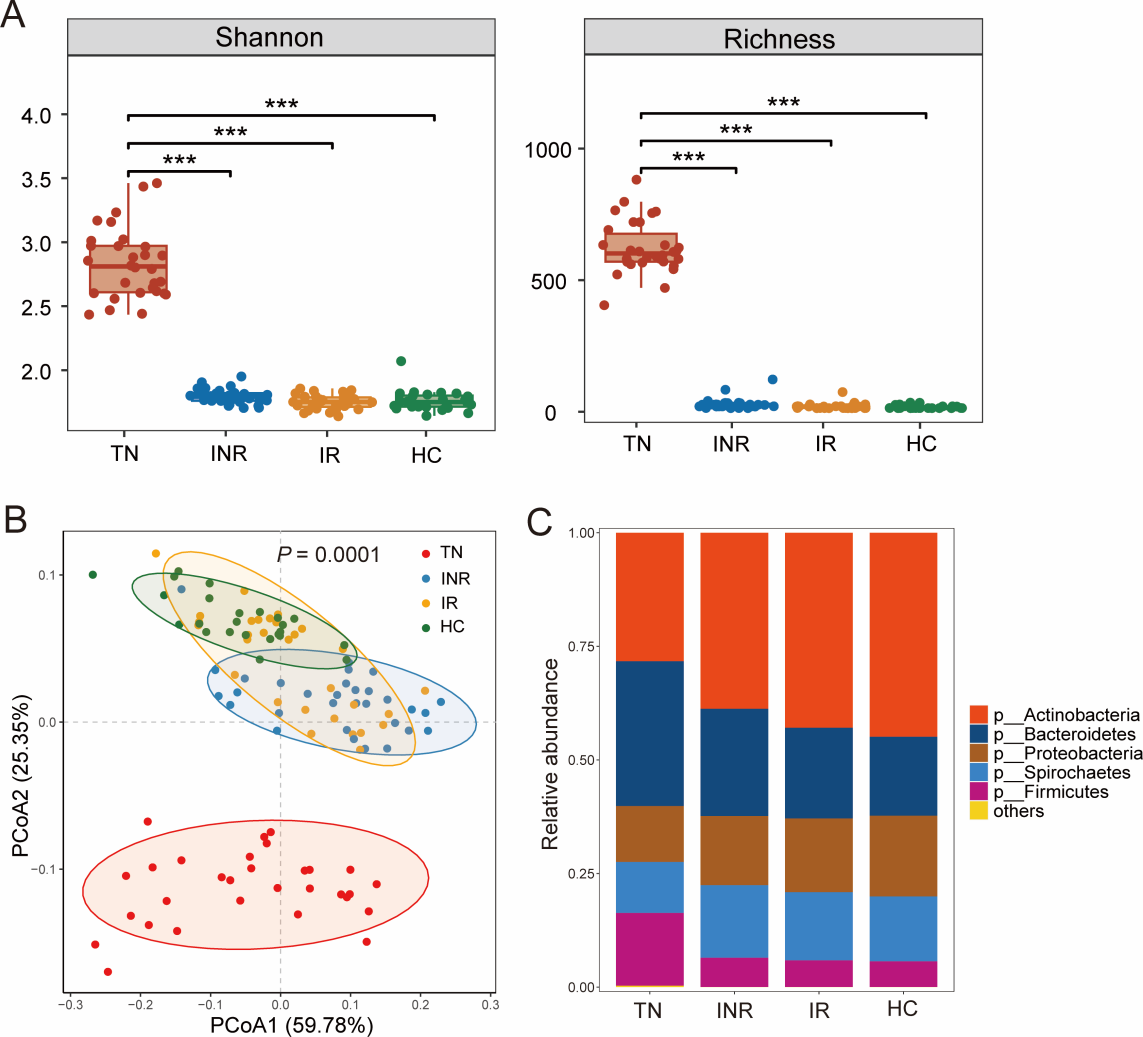
Taxonomic analysis of the blood microbiota of study subjects. (**A**) The Shannon index and richness analysis of blood microbiota in TNs, INRs, IRs, and HCs. *P < 0.05, **P < 0.01, ***P < 0.001. (**B**) Principal coordinates analysis (PCoA) of blood microbiota composition using Bray-Curtis dissimilarities in TNs, INRs, IRs, and HCs (*P* = 0.0001, analysis of similarity). (**C**) Average relative abundances of microbial phyla detected in blood from TNs, INRs, IRs, and HCs. Taxa are merged into the “Others” category if less abundant.

Alpha diversity was used to measure the abundance and evenness of the bacterial taxa within a community. We found that TN individuals exhibited significantly higher alpha diversity of blood microbiota than controls, as shown by the Shannon index (richness and evenness) and Richness index (richness only). ART significantly reduced alpha diversity in IRs and INRs (Fig. 2A). In contrast, TNs, INRs, and IRs (individuals infected with HIV) displayed lower Shannon and Richness indices in stool microbiota than controls, although there was no statistical difference (Fig. S1A).

To understand the impact of HIV infection on the distribution of blood microbiota, a principal coordinates analysis (PCoA) comparing samples based on Bray-Curtis differences revealed significantly different blood microbial compositions at the species level between the TN and HC groups. Interestingly, we also observed a significant gradient between TNs, INRs, IRs, and HCs [*P* = 0.0001, analysis of similarity (ANOSIM); Fig. 2B], with most IR samples overlapping with HC samples, while INR samples overlapped less with HCs and were closer to TNs. This grading suggests that the blood microbial distribution in patients with INRs is still relatively different from that in HCs.

Subsequently, we explored the composition of the blood microbiota and the effect of HIV infection. A graphical representation of the relative proportions of units at the phylum taxonomic level present in the study samples was performed. Blood was mainly composed of *Actinobacteria*, *Bacteroidetes*, *Proteobacteria*, *Spirochaetes*, and *Firmicutes*. Compared to HCs, *Actinobacteria* and *Proteobacteria* decreased, whereas *Bacteroidetes* and *Firmicutes* increased in the TNs. ART reduced this difference but did not restore it to normal levels, and the INRs differed more from that of HCs (Fig. 2C). Stacked bar plots represent the relative proportions of taxonomic units at other taxonomic levels (class, order, family, genus, and species in Fig. S2A through E, respectively). Interestingly, the gut microbiota in feces consisted mainly of the phyla *Bacteroidetes*, *Firmicutes*, and *Proteobacteria*, with a smaller proportion of *Actinobacteria*. The relative abundance of *Bacteroidetes* increased and *Firmicutes* decreased in individuals infected with HIV (Fig. S1B). Therefore, the increased relative abundance of the *Bacteroidetes* and *Firmicutes* phyla in the blood indicates that the intestinal lumen may be a source of these bacteria, which are then translocated into the blood.

### Identification of specific species associated with disease state and immune response

**Fig 3:**
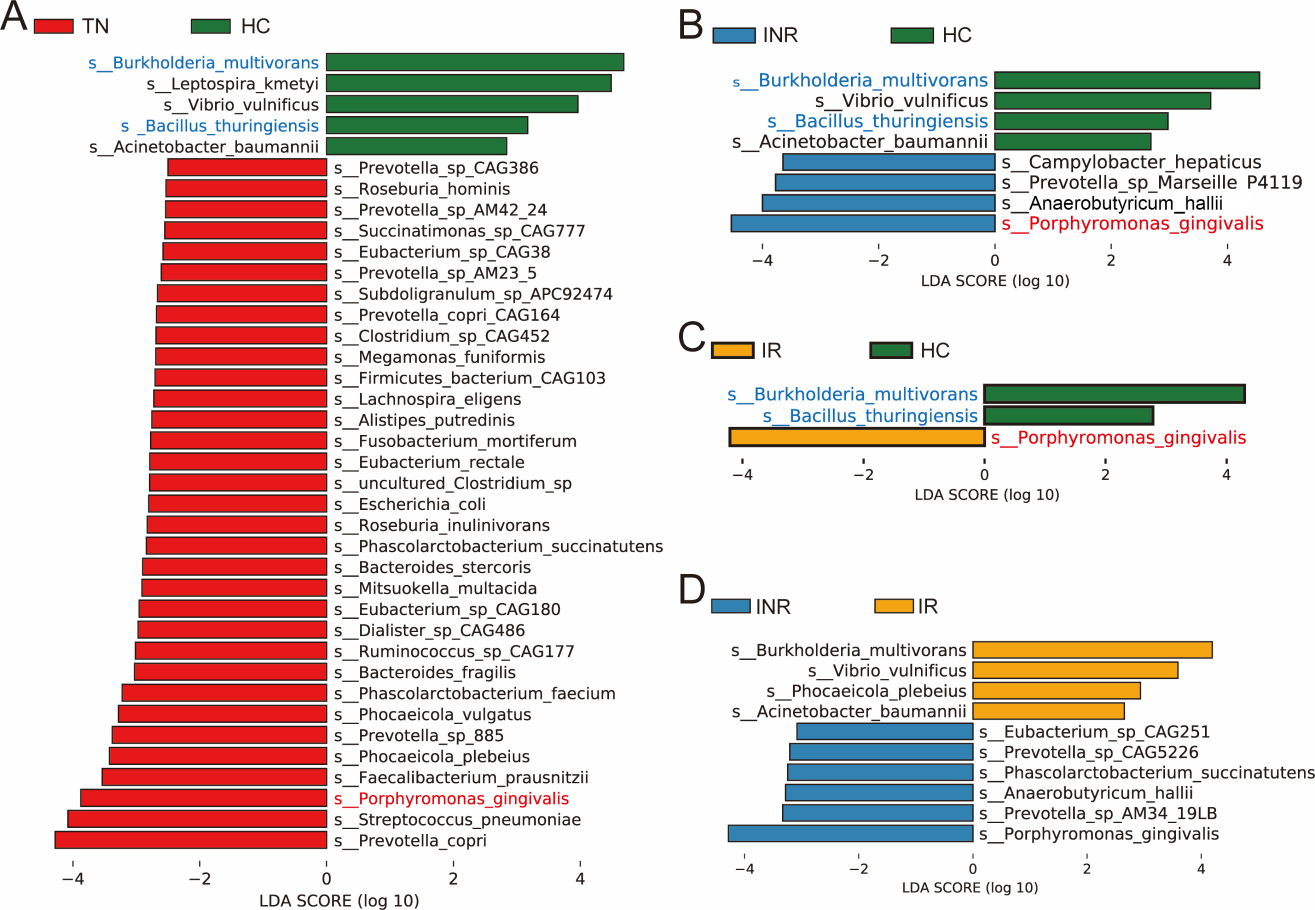
Differential enrichment of microbes in every two groups. Linear discriminant analysis effect size analysis of discriminant taxa in the blood microbiota of pairwise combinations of TNs compared to HCs (**A**), INRs compared to HCs (**B**), IRs compared with HCs (**C**), and INRs compared to IRs (**D**). Compared to HCs, bacterial species with enriched abundance in individuals infected with HIV are marked in red, and enriched species in HCs are marked in blue. *P* < 0.05; LDA score >2.5.

To identify specific species associated with disease states, we performed linear discriminant analysis (LDA) and effect size (LEfSe) analysis (*P* < 0.05, LDA >2.5). At the species level, the histograms showed that *Prevotella copri, Streptococcus pneumoniae, Porphyromonas gingivalis, Faecalibacterium prausnitzii, Phocaeicola plebeius, Prevotella* sp. *885, Phocaeicola vulgatus, Phascolarctobacterium faecium, Bacteroides fragilis, Ruminococcus* sp*. CAG177,* and 23 other species were enriched in TNs compared to HCs, while *Burkholderia multivorans, Leptospira kmetyi, Vibrio vulnificus, Bacillus thuringiensis,* and *Acinetobacter baumannii* were absent (Fig. 3A). Among the 33 species of bacteria enriched in TNs, 31 (approximately 94%) species belonged to the phyla *Bacteroidetes* and *Firmicutes* (Table S1). After long-term ART, this type of biological abnormality was attenuated, and most of the microbiota in IRs and INRs could be restored to normal levels. However, *P. gingivalis, Anaerobutyricum hallii, Prevotella* sp*. Marseille P4119,* and *Campylobacter hepaticus* were enriched, whereas *B. multivorans, V. vulnificus, B. thuringiensis,* and *A. baumannii* were absent in INRs compared with HCs (Fig. 3B). In addition, compared with HCs, *P. gingivalis* was enriched, whereas *B. multivorans* and *B. thuringiensis* were deficient in IRs (Fig. 3C). As a result, we found that *P. gingivalis* was consistently relatively enriched, while *B. multivorans* and *B. thuringiensis* were consistently relatively deficient in individuals infected with HIV (TNs, INRs, and IRs) compared to HCs.

Subsequently, we searched for differentially abundant species in the blood microbiota of INRs and IRs. An LEfSe analysis was performed (*P* < 0.05, LDA >2.5), and eight differentially abundant species were found in INRs compared to IRs. As shown in Fig. 3D, there were six species enriched in INRs and four species enriched in IRs. *P. gingivalis, Prevotella* sp. *AM34 19LB, A. hallii, Phascolarctobacterium succinatutens, Prevotella* sp*. CAG5226,* and *Eubacterium* sp*. CAG251* were enriched, and *B. multivorans*, *V. vulnificus, P. plebeius,* and *A. baumannii* were deficient in INRs compared with IRs. We found that approximately 50% (3/6) of these species-enriched INRs belonged to the phylum *Bacteroidetes* and the others belonged to the phyla Firmicutes. *B. multivorans, V. vulnificus,* and *A. baumannii* enriched in IRs were all classified as *Proteobacteria* at the phylum level (Table S2).

**Fig 4:**
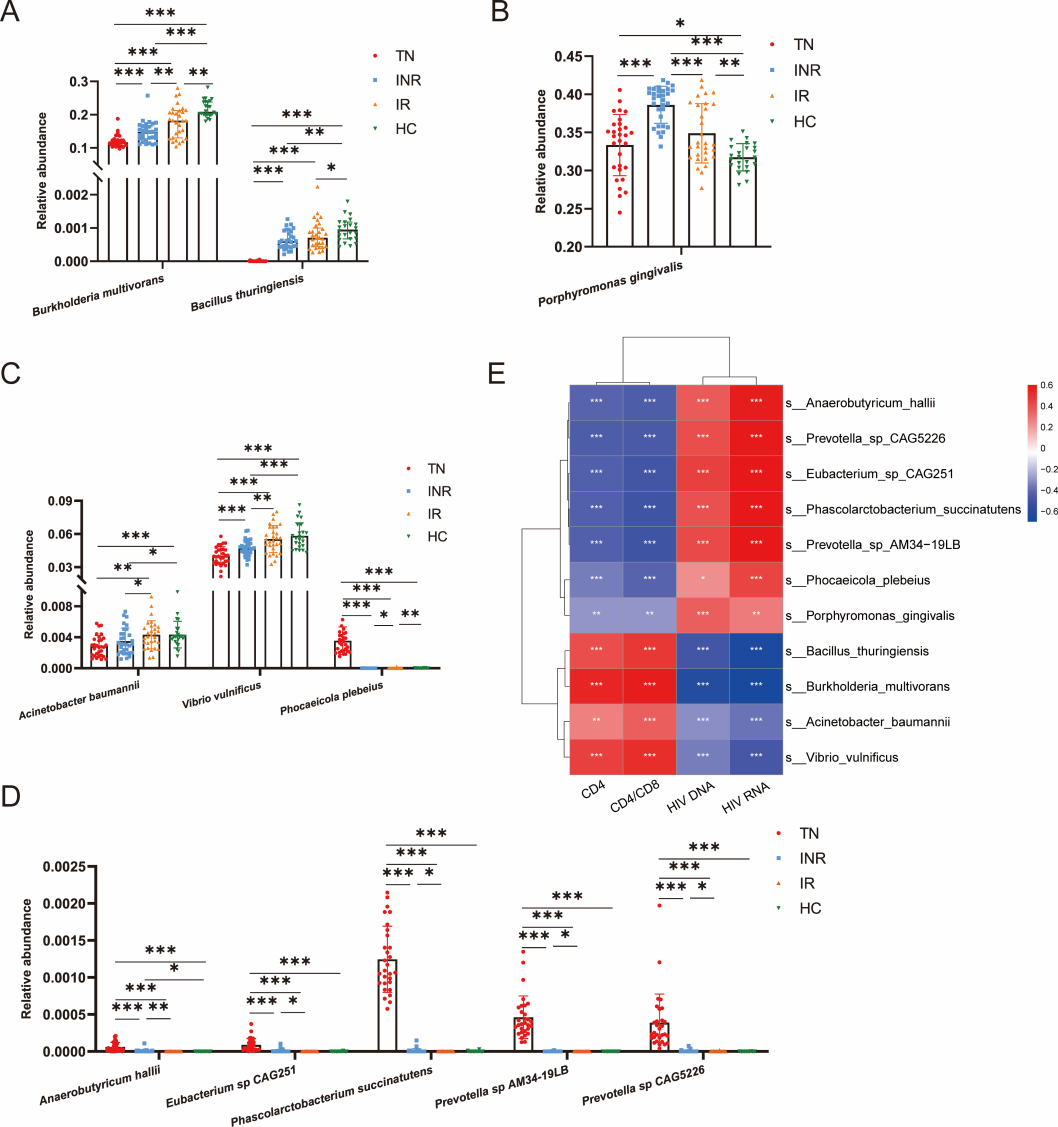
Differential bacterial species in blood associated with HIV disease status and immune recovery. (**A and B**) Species associated with disease states. Relative abundance of the two species enriched in HCs (**A**) and one species enriched in individuals infected with HIV (**B**). (**C and D**) Species associated with immune recovery. Relative abundance of the three species enriched in IRs (**C**) and five species enriched in INRs (**D**). Each dot represents a participant. *P < 0.05, **P < 0.01, ***P < 0.001. (**E**) Heatmap showing Spearman correlations between differential species selected above and clinical parameters and HIV reservoir indicators. Red and blue indicate positive and negative associations, respectively. *P < 0.05, **P < 0.01, ***P < 0.001.

We then visualized the relative abundance of the above 11 bacterial species in the four groups. The bar plots indicated that two species were consistently enriched in HCs (Fig. 4A), and the one species was enriched in individuals infected with HIV (Fig. 4B). The relative abundances of *B. multivorans* and *B. thuringiensis* were highest in HCs and lowest in TNs, whereas those in IRs were higher than those in INRs. However, *P. gingivalis* showed the opposite trend. Among the differential bacteria in INRs and IRs, three species were enriched in IRs (Fig. 4C), and five species were enriched in INRs (Fig. 4D), except *B. multivorans* and *P. gingivalis*. The relative abundances of *A. baumannii* and *V. vulnificus* were lowest in TNs and highest in HCs and were lower in INRs than in IRs. Interestingly, the relative abundances of *P. plebeius*, *A. hallii*, *E.* sp*. CAG251*, *P. succinatutens, P.* sp*. AM34-19LB,* and *P.* sp*. CAG5226* were high in TNs but very low in INRs, IRs, and HCs, some even close to zero, probably from translocation. Analysis of these 11 bacterial species in fecal samples did not reveal the same alteration in abundance as in blood (Fig. S3A through D).

To assess the possible impact of blood bacterial fractions on disease progression, we performed correlation analysis based on Spearman correlation with clinical indicators (CD4^+^ T-cell counts, CD4/CD8 ratio, HIV DNA, and RNA) for 11 bacteria selected from LEfSe analysis. We found that *B. multivorans, B. thuringiensis, V. vulnificus,* and *A. baumannii* showed significant positive correlations with the CD4/CD8 ratio and CD4^+^ T-cell counts, and significant negative correlations with HIV DNA and RNA. In addition, all six INRs-enriched bacteria and *P. plebeius* were negatively correlated with CD4^+^ T-cell counts and the CD4/CD8 ratio and positively correlated with HIV DNA and RNA (Fig. 4E).

### Plasma inflammation-related protein analysis

**Fig 5:**
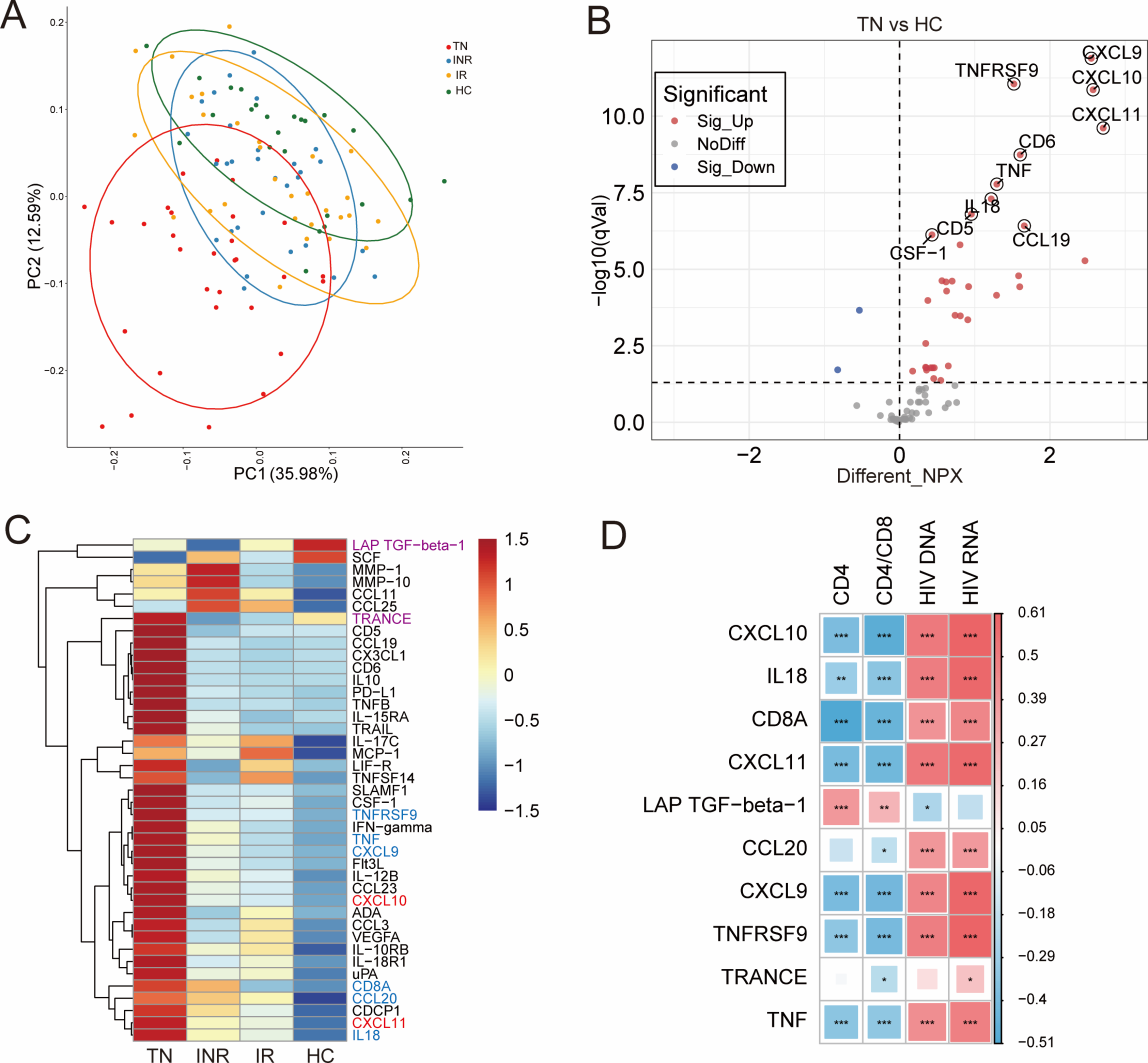
Alterations in inflammation-related proteins and correlation with clinical parameters in study design. (**A**) Principal component analysis showing the distribution of inflammation-related proteins in plasma in TNs (red), INRs (blue), IRs (yellow), and HCs (green). (**B**) Volcano plot of differentially expressed inflammation-related proteins in TNs compared to HCs. The red dots and blue dots represent the proteins with significantly higher and lower expression (adjusted *P* < 0.05) in the TN group, respectively. (**C**) Heatmap of differentially expressed inflammation-related proteins between the two different groups. Differential proteins that are elevated in TNs, INRs, and IRs compared to HCs are labeled in red, proteins that are elevated in INRs but not in IRs are labeled in blue, and proteins that are decreased in INRs but not in IRs are labeled in purple. (**D**) Heatmap showing Spearman correlations between differential inflammation-related proteins and clinical parameters and HIV reservoir indicators. Red and blue indicate positive and negative associations, respectively. *P < 0.05, **P < 0.01, ***P < 0.001.

To investigate the clinical significance of plasma inflammation-related proteins in HIV-infected patients, we measured 92 different inflammation-related proteins using the Olink multiplex inflammation assay panel. The expression profiles based on all proteins were visualized using the principal component analysis (PCA) downscaling method, which showed that the protein distribution was different and distinguishable between the four groups (Fig. 5A). We calculated the mean difference in the expression of each protein in the four groups of samples and compared their statistical significance based on statistical analysis. Thirty-seven inflammation-associated proteins were significantly different in TNs compared to HCs; 35 proteins were elevated, and 2 proteins were decreased (Fig. 5B; Table S3). Fourteen inflammation-related proteins differed in INRs compared to HCs; 12 proteins were elevated, and 2 proteins were decreased (Fig. S4A; Table S4). Compared to HCs, three inflammation-related proteins were different, and all were elevated in IRs (Fig. S4B; Table S5). As a consequence, a total of 41 inflammation-related proteins were significantly different in individuals infected with HIV compared to HCs (including TNs versus HCs, IRs versus HCs, and INRs versus HCs). Of these, two differential inflammation-related proteins were included in INRs and IRs (Table S6). In addition, the NPX expression levels of these inflammation-associated proteins were represented by a visual heatmap. Compared with HCs, CXCL10 and CXCL11, marked in red in Fig. 5C, are elevated in individuals infected with HIV (TNs, INRs, and IRs). Box plots showed that they were lowest in HCs and then increased in the order of IRs, INRs, and HCs (Fig. S5A). LAP TGF-β1 and tumor necrosis factor-related activation-induced cytokine (TRANCE) marked in purple are decreased, and TNFRSF9, TNF, CXCL9, CD8A, CCL20, and IL18 marked in blue are elevated in INRs, but there was no significant difference in IRs (Fig. 5C). Box plots revealed the expression of NPX in the four groups (Fig. S5B and C).

Moreover, to explore the relationship between inflammation-related proteins and clinical indicators, we correlated the 10 proteins most associated with the above disease progression with CD4^+^ T-cell counts, CD4/CD8 ratio, HIV DNA, and HIV RNA. The results showed that CXCL10, IL18, CD8A, CXCL11, CXCL9, TNFRSF9, and TNF were negatively correlated with CD4^+^ T-cell counts and the CD4/CD8 ratio but positively correlated with HIV DNA and RNA. CCL20 and TRANCE were weakly negatively correlated with the CD4/CD8 ratio and positively correlated with HIV RNA levels. Unlike other inflammation-related proteins, LAP TGF-β1 was positively correlated with CD4^+^ T-cell counts and the CD4/CD8 ratio and negatively correlated with HIV DNA (Fig. 5D).

### Relationship between blood bacterial fractions and inflammation-related proteins

**Fig 6:**
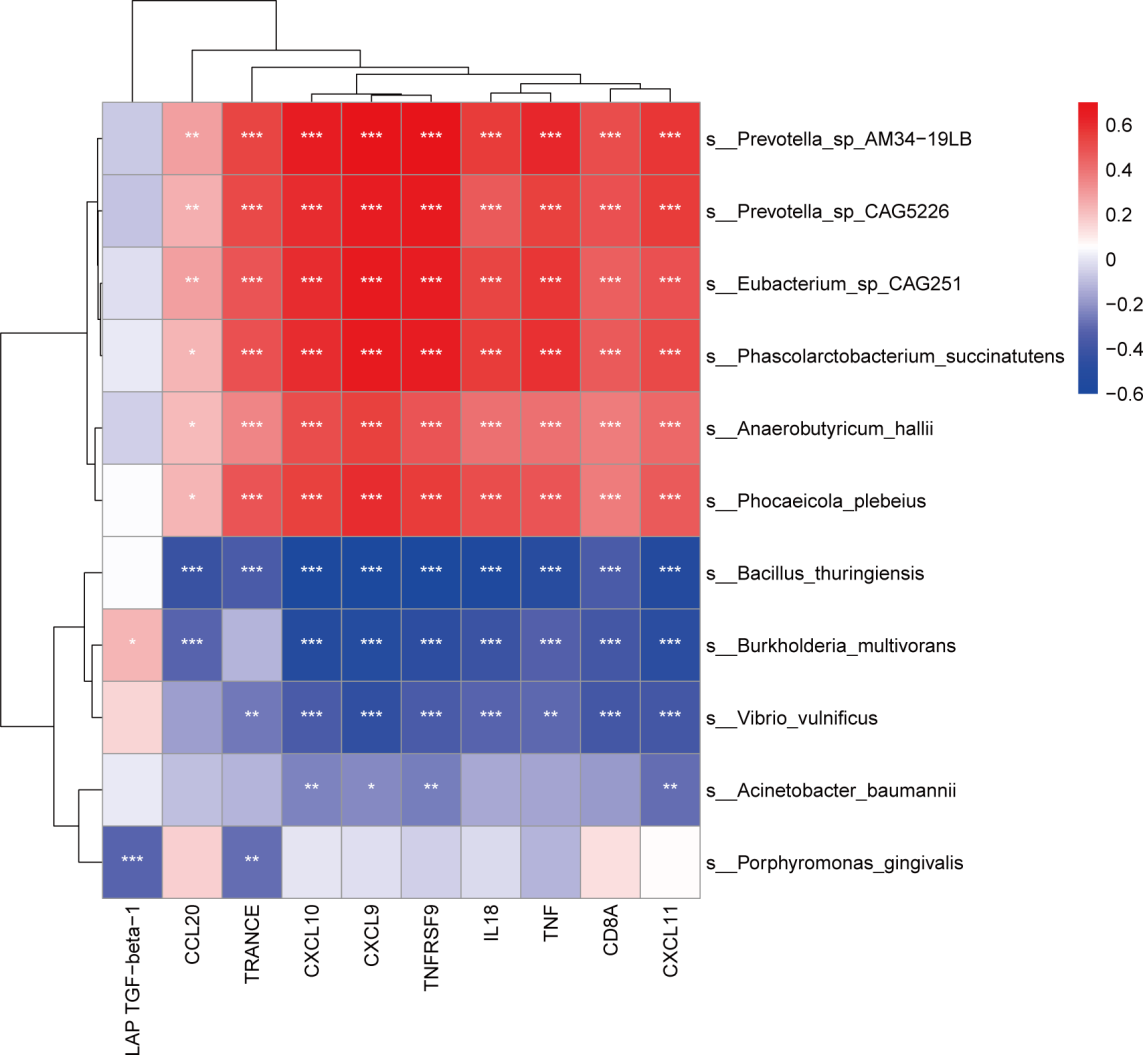
Correlations between differential bacterial species and inflammation-related proteins in study subjects. The heatmap shows Spearman correlations between the differentially abundant species and inflammation-related protein-associated disease states and immune recovery. Red and blue indicate positive and negative associations, respectively. *P < 0.05, **P < 0.01, ***P < 0.001.

To investigate the relationship between the blood microbiota associated with disease progression and inflammation-related proteins, we examined 11 bacterial species and 10 inflammation-associated protein correlations based on the Spearman coefficient (Fig. 6). The results demonstrated that inflammation-related proteins, except LAP TGF-β1, were positively correlated with *P. plebeius, A. hallii, E.* sp*. CAG251, P. succinatutens, P.* sp*. AM34-19LB,* and *P.* sp*. CAG5226* and negatively correlated with *B. thuringiensis. B. multivorans* was positively correlated with LAP TGF-β1 and negatively correlated with other proteins, excluding TRANCE. *V. vulnificus* was adversely correlated with TRANCE, CXCL10, CXCL9, TNFRSF9, IL18, TNF, CD8A, and CXCL11. *A. baumannii* was inversely correlated with CXCL10, CXCL9, TNFRSF9, and CXCL11. Interestingly, *P. gingivalis* was negatively correlated with LAP TGF-β1 and TRANCE. Accordingly, *P. gingivalis* is more likely to promote disease progression by inhibiting the expression of LAP TGF-β1 and TRANCE.

## DISCUSSION

In this study, we investigated the characteristics of the blood microbiota and inflammation-related proteins in individuals infected with HIV with different disease states and analyzed the relationship between these microbes and disease progression. We found that alpha diversity in the blood of individuals infected with HIV increased, particularly in TNs, whereas the diversity of gut microbes was reduced in stool samples. Accordingly, community PCoA showed differences in blood microbiota composition among TNs, INRs, IRs, and HCs, especially between TN and other groups, suggesting that ART partly repaired damaged intestinal epithelial integrity and reversed microbial translocation. The overall blood bacterial profile was dominated by the phyla *Actinobacteria*, *Bacteroidetes*, *Proteobacteria*, and *Firmicutes*. Furthermore, the number of inflammation-related proteins was significantly higher in TNs than in HCs, and INRs had more abnormal inflammation-related proteins than IRs. Among the differential inflammation-related proteins, the level of most inflammation-related proteins was higher in individuals infected with HIV, inversely correlated with CD4^+^ T-cell counts, and positively correlated with HIV viral reservoir indicators.

Previous studies have shown that reduced gut microbiota diversity is deleterious to the stability of the entire ecosystem and is associated with decreased CD4^+^ T-cells and increased T-cell activation (22, 23). An increase in gut microbial diversity and abundance is usually considered a more stable microbial community in the gut and more conducive to disease recovery. However, the impact of the increased diversity of blood microbes is different. Although there is rising evidence of the presence of microbes in the blood of healthy individuals, their species and numbers are significantly smaller than those of the gut microbiota (24 – 28). In the early stages of HIV infection, massive depletion of gastrointestinal CD4^+^ T-cells, especially Th17 cells, and concomitant structural damage to the epithelium lead to a significant increase in intestinal permeability and the entry of microbes and their products into the body circulation through the damaged mucosal barrier (19, 29 – 31). In the study of blood microbial composition, a significant increase was found in the TN group for *Bacteroidetes* and *Firmicutes*, which are the main phyla of the gut microbiota composition, suggesting a possible source of blood microbes with intestinal microbiota translocation. Blood microbiota diversity decreased substantially after ART but still did not return to normal levels, suggesting that microbial translocation persisted despite ART, which may be related to the persistence of intestinal damage (32, 33). Increased microbial diversity and altered composition affect the degree of CD4^+^ T-cell restoration and contribute to persistent inflammation (20, 21). This also responds to the fact that the blood microbiota not only reflects the microbial composition in the gut but also actively participates in the pathogenesis of HIV infection. Recent studies support this conclusion by demonstrating the importance of the blood microbiota in the development of diabetes, colon cancer, alcoholic hepatitis, and major depression in the general population (25, 26, 34, 35), possibly confirming the role of blood microbiota in non-AIDS-related comorbidities (36).

In addition to the increased diversity of the blood microbiota found in patients infected with HIV, the composition of the blood microbiota was also disrupted. Community PCoA highlighted differences in blood microbiota composition between TNs, INRs, IRs, and HCs. Based on metagenomic sequencing, the overall blood bacterial profile was dominated by the phyla *Actinobacteria*, *Bacteroidetes*, *Proteobacteria*, and *Firmicutes*, which are consistent with the dominant phyla found in most blood studies (21, 34, 37). At the species level, we found that many blood microbes in the TNs were disordered, and most of this disorder was corrected after receiving ART. However, some microbes differed significantly between the INR and IR groups and the HC group. We found that *P. gingivalis* was consistently enriched in individuals infected with HIV compared to HCs during different disease stages of HIV infection. *P. gingivalis* is a gram-negative rod-shaped specialized anaerobic bacterium that derives metabolic energy for growth from proteolytic products, heme, and vitamin K (38). It is an oral pathogen and, in addition to being a pathogen of periodontitis, *P. gingivalis* is involved in various systemic diseases such as cardiovascular disease, Alzheimer’s disease, and rheumatoid arthritis (39 – 41). Xie et al. found that *P. gingivalis* can mediate receptor-independent HIV-1 entry into the epithelial cells. Invasive bacteria and their outer membrane vesicles that interact with HIV-1 can mediate the transfer of HIV through the mucosa, establish mucosal transmission of HIV-1, and enhance HIV-1 infectivity (42, 43). Analysis of the presence of different bacteria in IRs and INRs, along with the observation that *P. gingivalis* was enriched in INRs, indicated that this bacterium may also play an important role in immune reconstitution. Interestingly, the other differential bacteria enriched in INRs were mostly present only in samples from the TN group and were absent in most samples from the other groups. These bacteria belong to the phyla *Bacteroidetes* and *Firmicutes* and may originate from intestinal microbial translocation. After receiving ART, disruption of the intestinal mucosal barrier was largely restored, and translocations were reduced. Nevertheless, the abundance of translocated bacteria varied owing to different degrees of mucosal recovery, and these translocated bacteria were negatively correlated with CD4^+^ T-cell counts and positively correlated with viral reservoir size. In particular, several studies have found *Prevotella* to be strongly associated with disease progression in HIV infection (44 – 46). The negative correlation of these bacteria with CD4^+^ T-cell counts and the CD4/CD8 ratio and the positive correlation with HIV viral reservoirs were detrimental to disease recovery.

In addition, we found that *B. multivorans* and *B. thuringiensis* were enriched in HCs compared to individuals infected with HIV. *B. multivorans*, *A. baumannii,* and *V. vulnificus* were enriched in IRs compared to INRs. *B. multivorans* kills pests and diseases by inhibiting fungal growth (47). *B. thuringiensis* (*Bt*) has become the main microorganism used for biological control. It contains chitinases that have an affinity for polymers and can degrade them, resulting in antimicrobial and immunomodulatory effects in the healthcare field (48). Another promising area is the potential of *Bt* proteins against cancer cells, with cytotoxic effects on cells altered by certain cancers, such as hepatocellular carcinoma (HepG2) and cervical cancer (HeLa) cells. This indicates the potential of microbes and new opportunities for future applications (49, 50). *A. baumannii* and *V. vulnificus* are gram-negative conditional pathogens, and a minor number of bacteria are necessary to maintain microecosystem stability (51, 52). It is worth noting that for these differential bacteria, trends in the feces were not the same as those in the blood. It is not plausible to use bacteria in feces to reflect bacterial changes in the blood, which are substantially less than those in feces, and the host is more sensitive to slight changes in blood bacteria, increasing the impact on disease progression. However, this remains hypothetical and must be tested in appropriately designed studies.

In this study, we found that species enriched in TNs and INRs were positively correlated with HIV DNA and RNA, whereas species enriched in HCs and IRs were negatively correlated with HIV DNA and RNA. This implicates that the HIV reservoir is closely related to blood microbiota. Previous studies have shown that the gut is one of the viral reservoirs (53, 54). A study found that the ratio of *Bacteroidales*/*Clostridiales* was inversely correlated with viral reservoir size (55). The relationship between blood microbes and HIV reservoirs has not been reported in current studies. Although there is evidence that the gut and blood microbiota composition have a strong effect on the host immune system (20, 56), it remains worthwhile to investigate how the microbial community interacts with the viral reservoir and whether it can influence the HIV reservoir through immune cell metabolism and proliferation (57 – 59). Inflammation is a hallmark of disease progression in response to HIV infection. We found that the number of inflammatory proteins was significantly higher in the TNs than in the HCs, and this difference did not fully normalize after ART. INRs have more abnormal inflammation-related proteins than IRs do. Among these differential inflammation-associated proteins, the levels of most were higher in individuals infected with HIV, inversely correlated with CD4^+^ T-cell counts, and positively correlated with HIV viral reservoir indicators. These inflammatory proteins mostly exert pro-inflammatory effects, which are consistent with previous findings. Yin et al. found that increased plasma CXCL9, CXCL10, and CXCL11 measured during primary HIV-1 infection predicted long-term HIV disease prognosis in men who have sex with men (MSM) and has the potential as a novel biomarker for clinical use (60). IL-18 may affect CXCL9, CXCL10, and CXCL11 by driving IFN-γ secretion (61). CCL20 can promote the efficient integration of HIV-1 in resting CD4^+^ T-cells by binding to CCR6 displayed on these cells (62). Therefore, blocking chemokine pathways, including CCR6/CCL20, maybe a possible route for therapeutic interventions to prevent viral transmission to key immune loci. CD8A is considered a marker molecule for killer T-cells and enhances CD8^+^ T-cell responses by acting as a co-receptor for TCRs (63). TGF-β1 levels are increased in the plasma and tissues of individuals infected with HIV. The TNF/TNFR pathway is involved in immune activation and viral reservoirs in HIV infection (64). Therefore, modulation of the TNF/TNFR pathway by novel therapeutic approaches could limit immune activation and reduce the size of the HIV reservoir in patients with undetectable viremia treated with ART (65). Notably, LAP TGF-β1 was reduced in individuals infected with HIV and had an anti-inflammatory effect. It was positively associated with CD4^+^ T-cells and the CD4/CD8 ratio and inversely associated with viral reservoir indicators. TGF-β1 is a homeostatic factor that maintains immune system homeostasis and orchestrates complex tissue repair after organ injury or infection. TGF-β1 also controls the proliferation, survival, and reactivity of naïve CD4^+^ and CD8^+^ T-cells in the peripheral blood, which are essential for maintaining peripheral immune homeostasis and T-cells defending against immune change (66). TGF-β1 normally inhibits TCR-mediated T-cell activation, including the suppression of Th1 cells, Th2 cells, and cytotoxic T lymphocytes. In contrast, TGF-β1 supports the differentiation and maintenance of peripheral Tregs, Th9, and Th17 cells (67). TGF-β1 negatively regulates B-cell survival, proliferation, immunoglobulin (Ig) synthesis, and IgG class switching but promotes IgA antibody production and plays an important role in mucosal immunity (68, 69). Interestingly, TRANCE was lower in the INR group than in the other groups. This is consistent with previously reported results (70 – 72). Lower levels of TRANCE were reported to be an independent predictor of nontraumatic fractures, suggesting an effect on osteoclastogenesis (73), affecting bone marrow cell development and leading to a decrease in bone marrow cells, CD34^+^ hematopoietic progenitor cells, and granulocytes (74).

Translocation of the gut microbiota to the blood is one of the causes of systemic immune activation during HIV infection (20). Our study explored the relationship between blood microbiota and inflammation-related proteins. Blood microbiota was found to be positively correlated with inflammation-related proteins (positively correlated with CD4^+^ T-cells and negatively correlated with HIV reservoir), suggesting that it may be the blood microbiota that influences disease progression in individuals infected with HIV by causing an inflammatory response that leads to alterations in inflammation-related proteins.

This study had several limitations. First, the frequency of MSM in our HIV group was higher than that in the control group, which may have influenced the results. In addition, the mechanism by which the blood microbiota affects inflammation-related proteins and thus immune recovery needs further analysis, as this association does not necessarily imply causality, and further animal experiments are needed to verify this. Finally, future longitudinal or prospective studies are needed to reveal that comprehensive medical records including lifestyle habits, intestinal inflammation, and intestinal injury may provide further evidence for our hypothesis.

In conclusion, this study characterized blood microbiota abnormalities and inflammation-related proteins in individuals infected with HIV. Blood microbial *P.* sp*. CAG:5226, E.* sp*. CAG:251, P. succinatutens, A. hallii, P.* sp*. AM34-19LB, P. plebeius,* and *P. gingivalis* were positively correlated with pro-inflammatory proteins and HIV DNA and RNA and negatively correlated with anti-inflammatory proteins, CD4^+^ T-cells, and the CD4/CD8 ratio. The opposite was true for the blood microbes *B. multivorans, B. thuringiensis, V. vulnificus,* and *A. baumannii*. These results will help identify effective microbial and immunotherapeutic strategies for HIV infection.

## MATERIALS AND METHODS

### Study population

We recruited 30 TNs with chronic HIV infection, 31 INRs (CD4^+^ T-cell counts < 350 cells/µL), and 30 IRs (CD4^+^ T-cell counts > 500 cells/µL) who had successfully received ART for more than 2 years with plasma HIV RNA below detectable levels. Controls were recruited from healthy non-HIV-infected individuals who accompanied patients to the clinic, medical students, or hospital staff to form an age-matched group. Exclusion criteria included co-infection with hepatitis B virus or hepatitis C virus, tuberculosis and other opportunistic infections, pregnancy, use of antibiotics, probiotics, or prebiotics within the past 1 month or diarrhea or digestive symptoms. The detailed characteristics of the participants are listed in Table 1. This study was approved by the Ethics Committee of the Fifth Medical Center of the PLA General Hospital. Each subject signed an informed consent form before enrollment.

### Sample collection

Peripheral blood samples were collected under sterile conditions and were immediately processed. The sample was then divided into two aliquots of 4 mL in a biosafety cabinet, and one of the tubes was stored in a −80°C refrigerator until DNA extraction. The other was centrifuged at 400 × *g* for 10 minutes to isolate the plasma, which was stored frozen at −80°C for further proteomic analysis. Peripheral blood mononuclear cells were isolated using density gradient centrifugation using Ficoll-Paque PLUS (17-1440-03, GE Healthcare).

The researchers distributed disposable sterile potty and tubes to the participants in advance. Participants first discharged the feces in a sterile potty, then washed their hands, put on disposable gloves, and took the middle part of the feces. Fecal samples from patients and HCs were freshly collected and frozen at −80°C within 4 hours after sampling.

### DNA extraction

All microbial DNA was extracted from whole blood and stool samples using the QIAamp DNA Blood Mini Kit (51106, Qiagen) and QIAamp PowerFecal Pro DNA Kit (51804, Qiagen), according to the manufacturer’s instructions. The concentration and purity of the extracted DNA were determined using TBS-380 (Turner Biosystems, Sunnyvale, CA, USA) and NanoDrop2000 (Thermo Scientific, Wilmington, DE, USA), respectively. DNA extraction quality was checked on a 1% agarose gel.

### DNA library construction and sequencing

Paired-end library was constructed using NEXTflexTM Rapid DNA-Seq (Bioo Scientific, Austin, TX, USA). Shotgun metagenomic sequencing was performed on DNBSEQ-T7 platform (MGI Tech Co., Ltd., Guangdong, China) using DNBSEQ-T7RS Reagent Kit (FCL PE150) version 2.0, according to the manufacturer’s instructions. All samples were paired-end sequenced with a 150-bp read length to a targeted data size of 10.0 Gb.

### Sequence quality control and genome assembly

The raw reads from metagenome sequencing were used to generate clean reads by removing adaptor sequences, trimming, and removing low-quality reads using fastp (version 0.20.0). The clean reads were mapped to the human hg38 reference genome using BWA (version 0.7.9 a) to identify and remove human host-originated reads.

These high-quality reads were then assembled into contigs using MEGAHIT (version 1.1.2), which use succinct de Bruijn graphs. Contigs with lengths greater than 300 bp were selected as the final assembly result.

### Gene prediction and taxonomy

Open reading frames in contigs were identified using MetaGene. A nonredundant gene catalog was constructed using CD-HIT (version 4.6.1) with 90% sequence identity and 90% coverage. Reads after quality control were mapped to the nonredundant gene catalog with 95% identity using SOAPaligner (version 2.21), and gene abundance in each sample was evaluated. Representative sequences of the non-redundant gene catalog were annotated based on the NCBI NR database using BLAST as implemented in DIAMOND version 0.9.19 with an e-value cutoff of 1e^−5^ using Diamond (version 0.8.35) for taxonomic annotations.

### Microbial community diversity analysis

Each sample’s alpha diversity (Shannon index) was calculated using the R package VEGAN (version 2.5.3) on the relative abundance of species. Species richness for all samples was estimated based on rarefied data. Beta diversities (Bray-Curtis dissimilarities) among samples were calculated using VEGAN based on the relative abundance of species.

### Plasma protein profiling

The OLINK inflammation panel of 92 proteins was used, which uses the proximity extension assay technology to obtain normalized protein expression (NPX) values for 92 proteins. (Olink Proteomics, Watertown, MA, USA; see Table S1 for an overview of immune markers, including their full names). Each antibody was labeled separately with unique proximity extension assay oligonucleotide probes, two separate and complementary sequences. NPX was calculated from the Ct values of the PCR readout. The analytical performance of each protein assay included in the panel was carefully validated based on specificity, sensitivity, dynamic range, precision, scalability, endogenous interference, and detectability (http://www.olink.com). After quality control, 67 proteins were included in the analysis.

### Detection of HIV DNA and RNA

Total cellular HIV DNA and RNA were extracted from peripheral blood mononuclear cells using QIAamp DNA Blood Mini Kit (51106, Qiagen) and HiPure Total RNA Plus Mini Kits (R4121-01, Magen), respectively. HIV DNA and RNA were quantified using the HIV DNA Quantitative Detection Kit (SUPI-1116, SUPBIO) and HIV RNA Quantitative Detection Kit (SUPI-0103, SUPBIO). Both kits were used according to the manufacturer’s instructions.

### Statistical analysis

Statistical analyses were performed using R Studio version 4.1.0 (R Studio, Boston, MA, USA), GraphPad Prism 8.0 (GraphPad Software, San Diego, CA, USA), and SPSS version 26.0 (SPASS, Chicago, IL, USA). Continuous variables are expressed as medians (IQRs), whereas categorical variables are presented as numbers (percentages). The Mann-Whitney *U* test (between two groups) and Kruskal-Wallis test (for more than two groups) were used to compare continuous data. To visualize the microbial community and functional profiles, as well as the protein distribution among the three groups, we calculated the between-sample diversity score (Bray-Curtis distance). PCA was used to visualize the separation. To test the difference in microbial composition between two or more groups, ANOSIM was employed based on Bray-Curtis dissimilarity. LEfSe analysis was used to identify the taxa or functional profiles most likely to explain the differences between two or three groups. An LDA score cutoff of 2.5 indicated a significant difference. Correlations were determined using the Spearman rank correlation test between two continuous variables. *P* < 0.05 was considered statistically significant. In addition, a Benjamini-Hochberg false-discovery rate-corrected *P* value was estimated.

## Data Availability

Sequences and metadata are available in NCBI under accession number PRJNA953845.

## AFTER EPUB

*[This article was published on 12 September 2023 with an error in the order of corresponding authors in the article PDF. The error was corrected in the current version, posted on 26 October 2023.]*

## SUPPLEMENTAL MATERIAL

Supplemental figure legends msystems.00467-23-s0001.docx Legends to Fig. S1 to S5.

Figure S1 msystems.00467-23-s0002.tif Taxonomic analysis of the gut microbiota of study subjects.

Figure S2 msystems.00467-23-s0003.tif Taxon relative abundance of blood microbiota at every taxonomic level.

Figure S3 msystems.00467-23-s0004.tif Expression levels of the species in feces corresponding to Figure 4.

Figure S4 msystems.00467-23-s0005.tif Differential inflammation-related proteins in immunological non-responders and immunological responders versus healthy controls.

Figure S5 msystems.00467-23-s0006.tif Inflammation-related proteins in plasma associated with HIV disease status and immune recovery.

Supplemental tables msystems.00467-23-s0007.docx Tables S1 to S6.

ASM does not own the copyrights to Supplemental Material that may be linked to, or accessed through, an article. The authors have granted ASM a non-exclusive, world-wide license to publish the Supplemental Material files. Please contact the corresponding author directly for reuse.
