# Supplementary material for: Abnormal blood microbiota profiles are associated with inflammation and immune restoration in HIV/AIDS individuals
Source: mSystems. 2023 Sep 12;8(5):e00467-23. doi: 10.1128/msystems.00467-23 (PMC10654078; doi:10.1128/msystems.00467-23)
Supplement: Supplemental figure legends — Legends to Fig. S1 to S5. [file msystems.00467-23-s0001.docx]

**Supplementary figure legend**

**Figure S1 Taxonomic analysis of the gut microbiota of study subjects.**

(A) The Shannon index and richness analysis of gut microbiota in treatment-naïve individuals (TNs), immunological non-responders (INRs), immunological responders (IRs), and healthy controls (HCs). **P* < 0.05, ***P* < 0.01, ****P* < 0.001. (B) Average relative abundances of microbial phyla detected in feces from TNs, INRs, IRs, and HCs. Taxa are merged into the “Others” category if less abundant.

**Figure S2 Taxa relative abundance of blood microbiota at every taxonomic level.**

Stacked column bar graphs depicting the taxonomic relative abundance of the blood microbiota at class (A), order (B), family (C), genus (D), and species (E) levels for each group.

**Figure S3 Expression levels of the species in feces corresponding to Figure 4.**

The histogram shows the relative abundance of 11 bacterial species: (A) corresponds to Figure 4A, (B) corresponds to Figure 4B, (C) corresponds to Figure 4C, and (D) corresponds to Figure 4D.

**Figure S4 Differential inflammation-related proteins in immunological non-responders (INRs) and immunological responders (IRs) versus healthy controls (HCs).**

(A) Volcano plot of differentially expressed inflammation-related proteins in INRs compared to HCs. The red and blue dots represent the proteins with significantly higher and lower expression (adjusted *P* < 0.05) in the INR group, respectively. Heatmap of differential inflammation-related protein expression per sample in INRs and HCs. (B) Volcano plot of differentially expressed inflammation-related proteins in IRs compared to HCs. The red and blue dots represent the proteins with significantly higher and lower expression (adjusted *P* < 0.05) in the IR group, respectively. Heatmap of differential inflammation-related protein expression per sample in IRs and HCs.

**Figure S5 Inflammation-related proteins in plasma associated with HIV disease status and immune recovery.**

(A) Expression levels of CXCL10 and CXCL11 were elevated in individuals infected with HIV. (B-C) Expression levels of (B) LAP TGF-β1 and TRANCE were elevated in immunological non-responders (INRs) and (C) CD8A, CXCL9, CCL20, IL18, TNF and TNFRSF9 in treatment-naïve individuals (TNs), INRs, immunological responders (IRs), and healthy controls (HCs). Data are expressed as the median (interquartile range, IQR). Each dot represents a participant. **P* < 0.05, ***P* < 0.01, ****P* < 0.001.
