# Supplementary figures and images for: Abnormal blood microbiota profiles are associated with inflammation and immune restoration in HIV/AIDS individuals
Source: mSystems. 2023 Sep 12;8(5):e00467-23. doi: 10.1128/msystems.00467-23 (PMC10654078; doi:10.1128/msystems.00467-23)

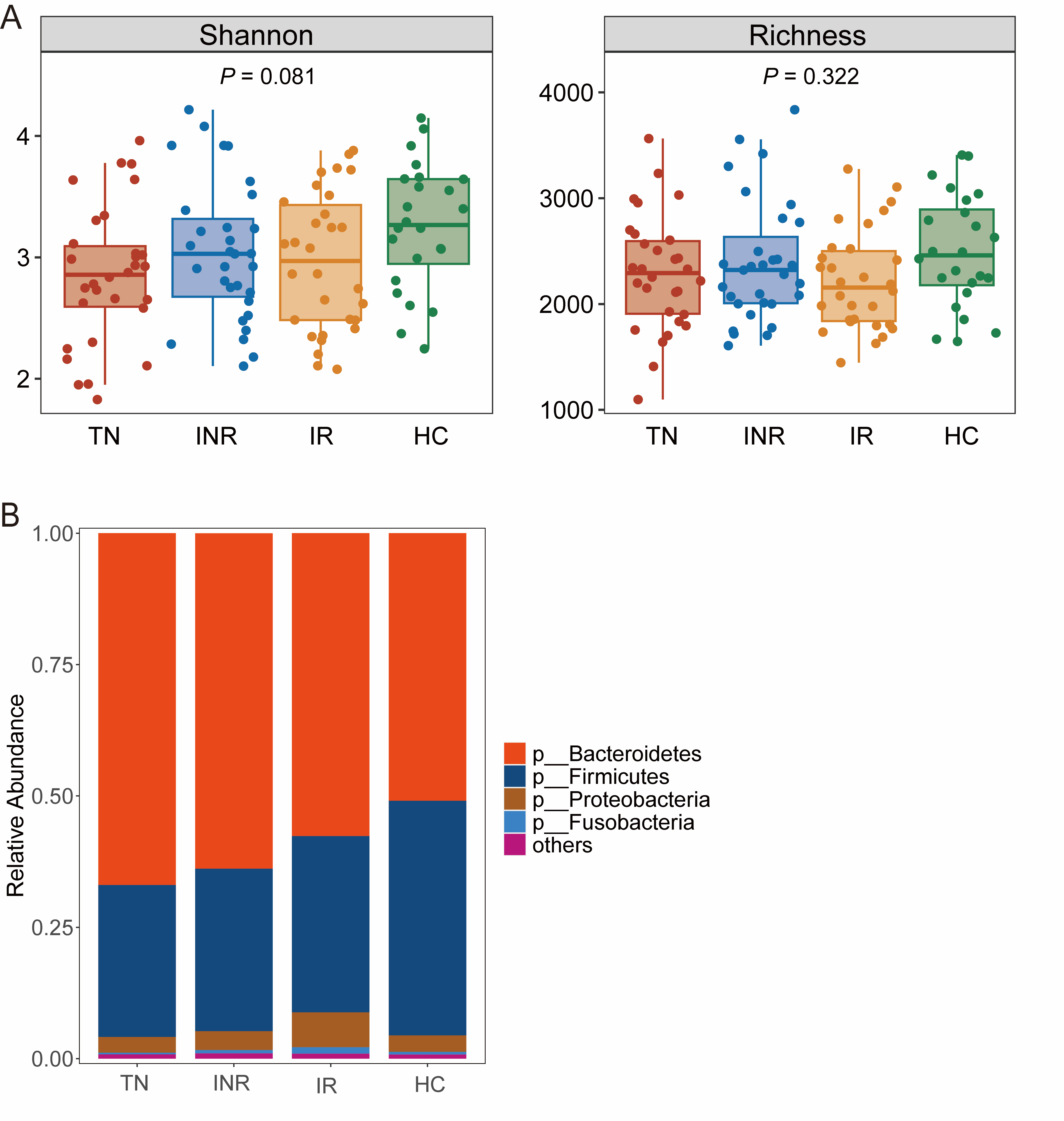

Supplement: Figure S1 — Taxonomic analysis of the gut microbiota of study subjects. [file msystems.00467-23-s0002.tif]

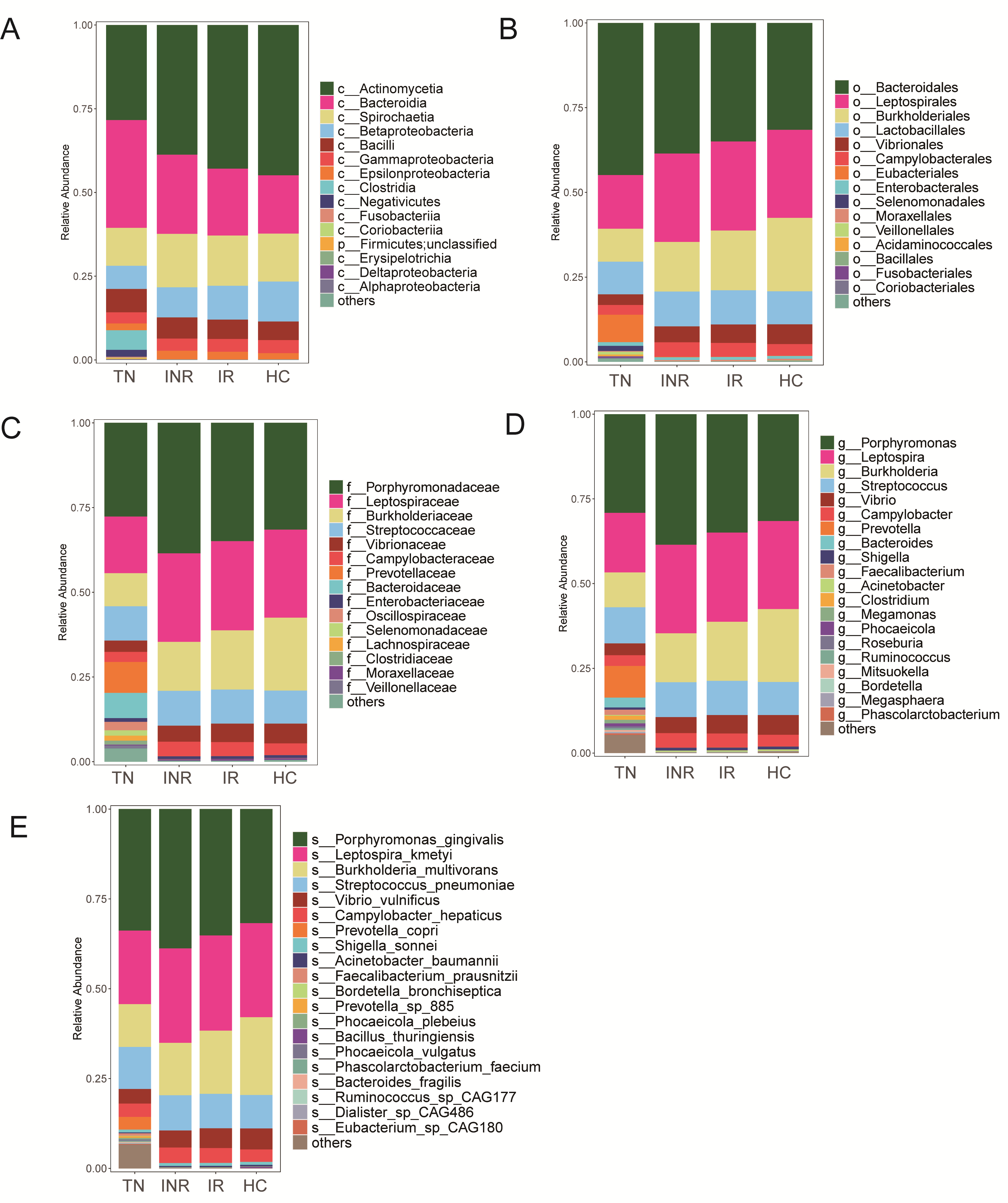

Supplement: Figure S2 — Taxon relative abundance of blood microbiota at every taxonomic level. [file msystems.00467-23-s0003.tif]

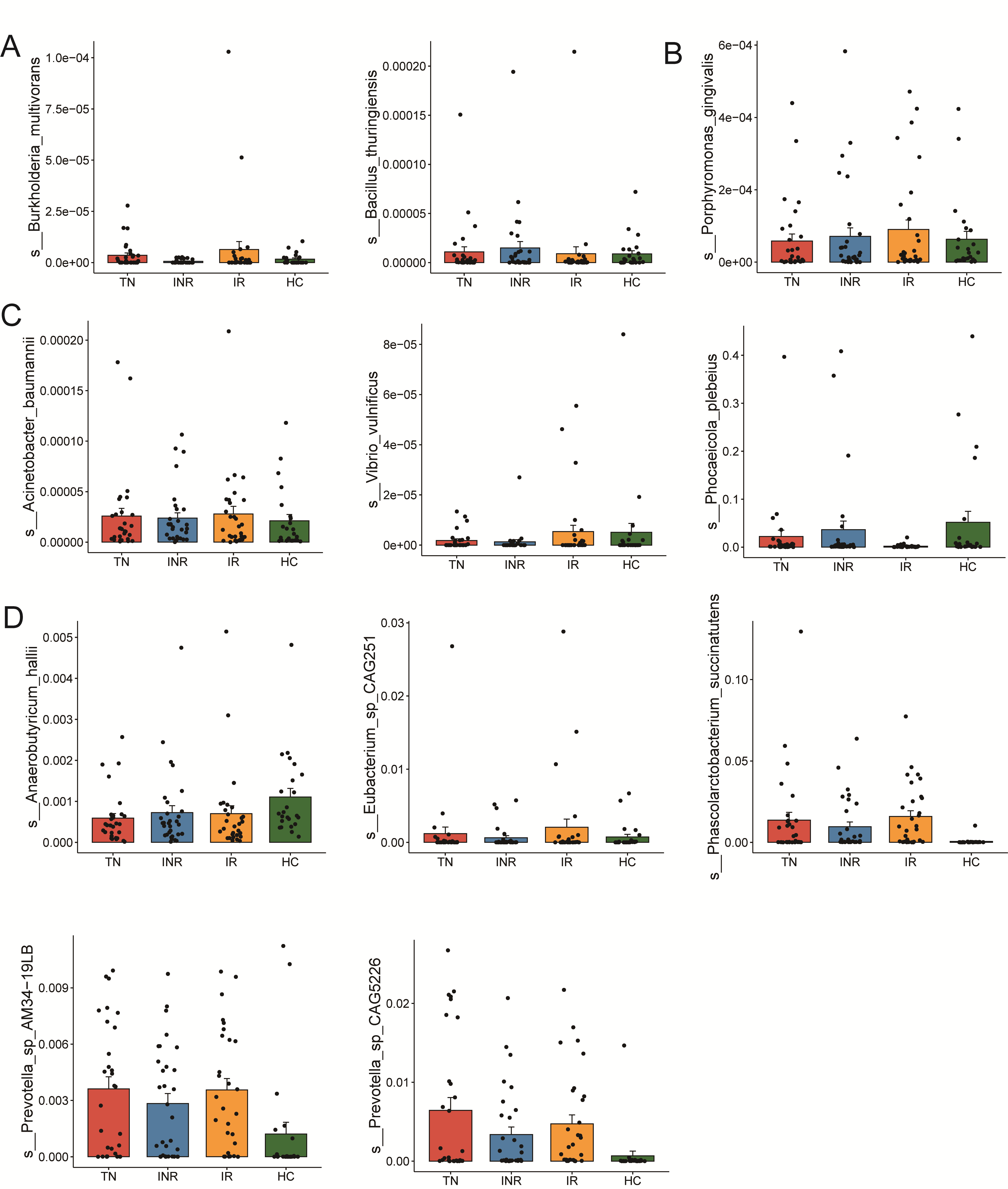

Supplement: Figure S3 — Expression levels of the species in feces corresponding to Figure 4. [file msystems.00467-23-s0004.tif]

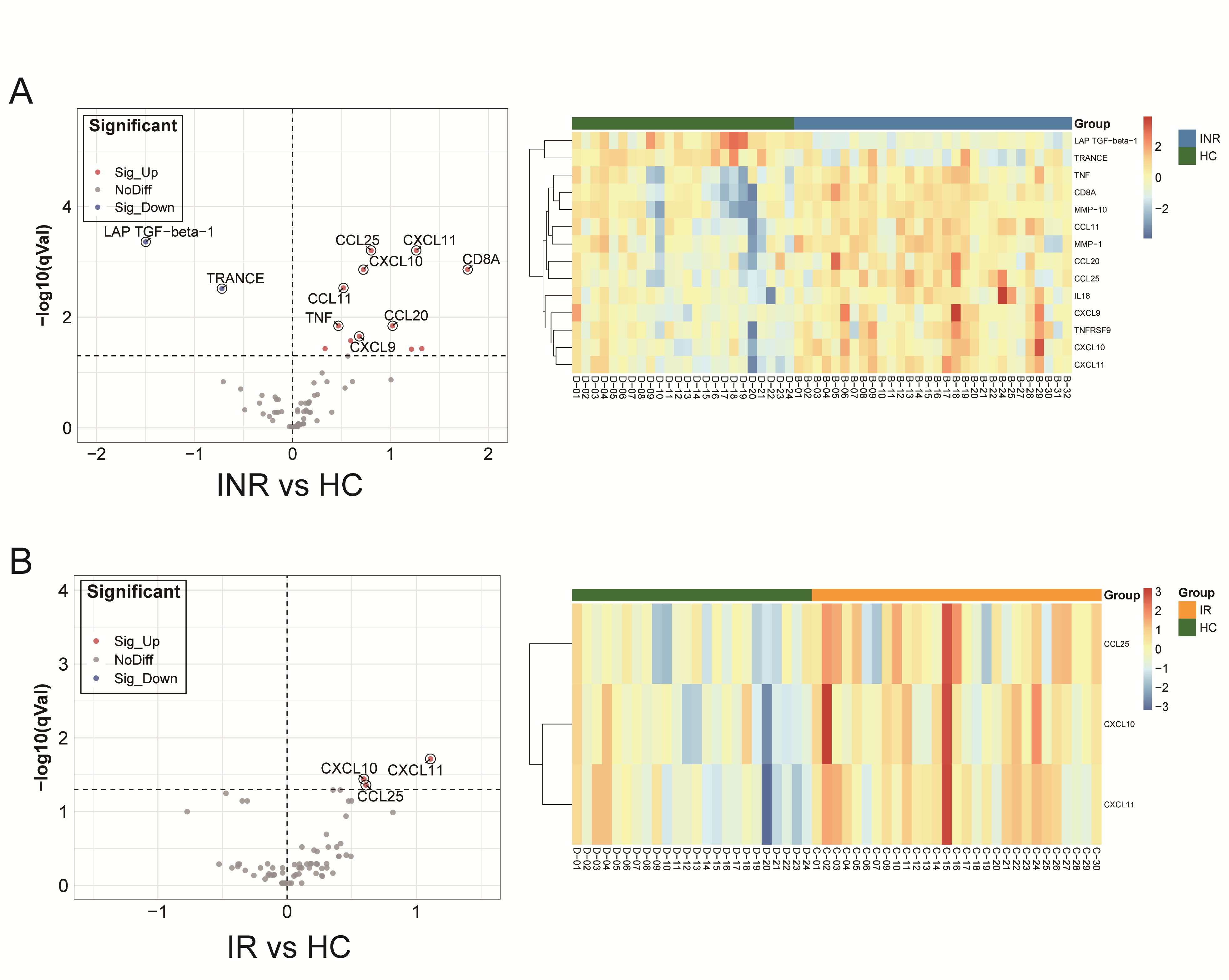

Supplement: Figure S4 — Differential inflammation-related proteins in immunological non-responders and immunological responders versus healthy controls. [file msystems.00467-23-s0005.tif]

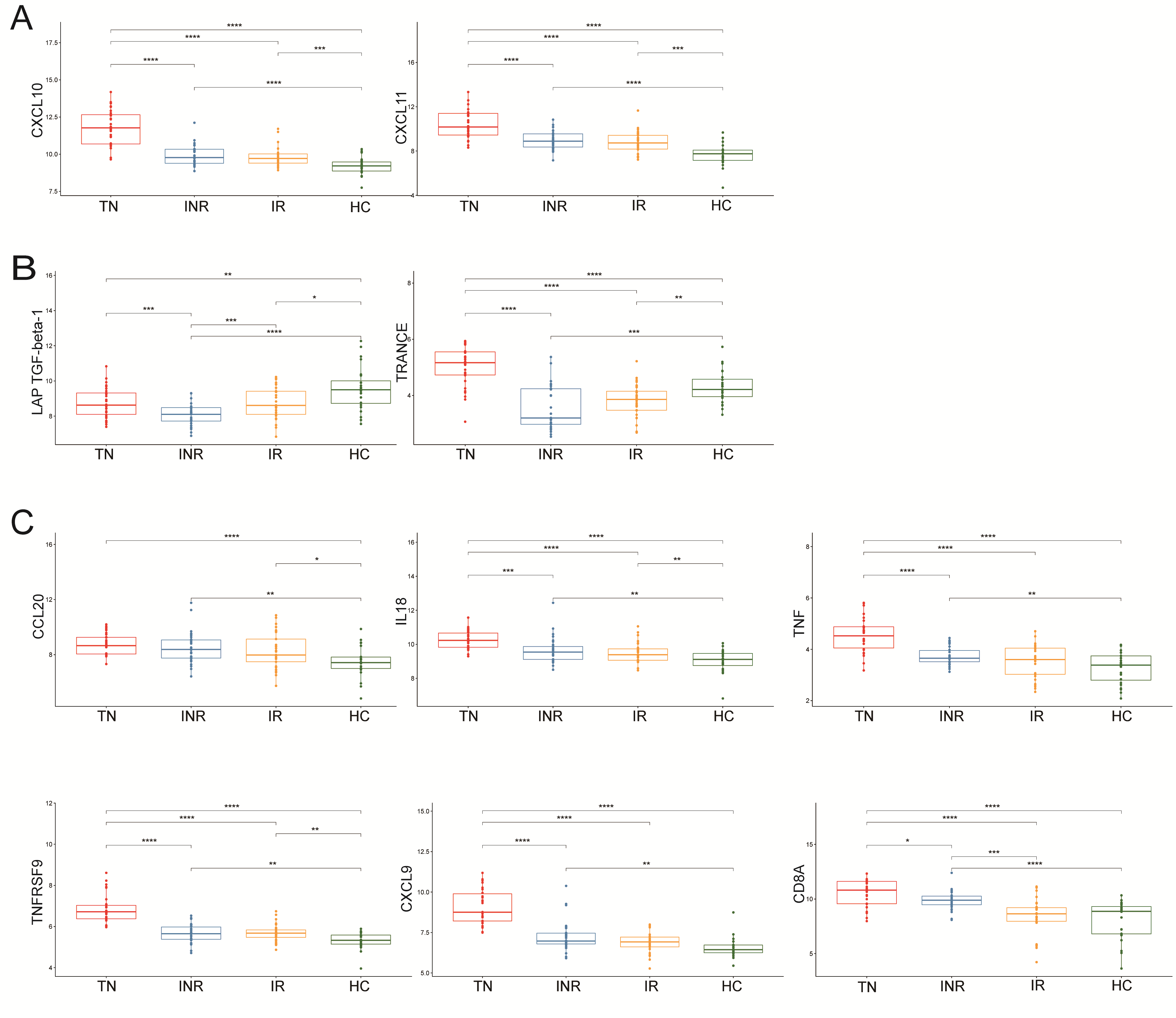

Supplement: Figure S5 — Inflammation-related proteins in plasma associated with HIV disease status and immune recovery. [file msystems.00467-23-s0006.tif]
