# Supplementary material for: Abnormal blood microbiota profiles are associated with inflammation and immune restoration in HIV/AIDS individuals
Source: mSystems. 2023 Sep 12;8(5):e00467-23. doi: 10.1128/msystems.00467-23 (PMC10654078; doi:10.1128/msystems.00467-23)
Supplement: Supplemental tables — Tables S1 to S6. [file msystems.00467-23-s0007.docx]

**Table S1**. The phylum and subsequent taxonomic levels of bacterium with LDA effect size (LEfSe) plots of pairwise analysis for treatment-naïve HIV-1-infected individuals and healthy controls

| Phylum | Class | Order | Family | Genus | Species |
| --- | --- | --- | --- | --- | --- |
| Proteobacteria | Betaproteobacteria | Burkholderiales | Burkholderiaceae | Burkholderia | Burkholderia multivorans |
| Spirochaetes | Spirochaetia | Leptospirales | Leptospiraceae | Leptospira | Leptospira kmetyi |
| Proteobacteria | Gammaproteobacteria | Vibrionales | Vibrionaceae | Vibrio | Vibrio vulnificus |
| Firmicutes | Bacilli | Bacillales | Bacillaceae | Bacillus | Bacillus thuringiensis |
| Proteobacteria | Gammaproteobacteria | Moraxellales | Moraxellaceae | Acinetobacter | Acinetobacter baumannii |
| Bacteroidetes | Bacteroidia | Bacteroidales | Prevotellaceae | Prevotella | Prevotella sp. CAG:386 |
| Firmicutes | Clostridia | Eubacteriales | Lachnospiraceae | Roseburia | Roseburia hominis |
| Bacteroidetes | Bacteroidia | Bacteroidales | Prevotellaceae | Prevotella | Prevotella sp. AM42-24 |
| Firmicutes | Clostridia | Eubacteriales | Oscillospiraceae | Ruminococcus | Ruminococcus sp. CAG:177 |
| Firmicutes | Clostridia | Eubacteriales | Eubacteriaceae | Eubacterium | Eubacterium sp. CAG:38 |
| Bacteroidetes | Bacteroidia | Bacteroidales | Prevotellaceae | Prevotella | Prevotella sp. AM23-5 |
| Firmicutes | Clostridia | Eubacteriales | Oscillospiraceae | Subdoligranulum | Subdoligranulum sp. APC924/74 |
| Bacteroidetes | Bacteroidia | Bacteroidales | Prevotellaceae | Prevotella | Prevotella copri CAG:164 |
| Firmicutes | Clostridia | Eubacteriales | Clostridiaceae | Clostridium | Clostridium sp. CAG:452 |
| Firmicutes | Negativicutes | Selenomonadales | Selenomonadaceae | Megamonas | Megamonas funiformis |
| Firmicutes | NA | NA | NA | NA | Firmicutes bacterium CAG:103 |
| Firmicutes | Clostridia | Eubacteriales | Lachnospiraceae | Lachnospira | Lachnospira eligens |
| Bacteroidetes | Bacteroidia | Bacteroidales | Rikenellaceae | Alistipes | Alistipes putredinis |
| Fusobacteria | Fusobacteriia | Fusobacteriales | Fusobacteriaceae | Fusobacterium | Fusobacterium mortiferum |
| Firmicutes | Clostridia | Eubacteriales | Lachnospiraceae | NA | Eubacterium rectale |
| Firmicutes | Clostridia | Eubacteriales | Clostridiaceae | Clostridium | uncultured Clostridium sp. |
| Proteobacteria | Gammaproteobacteria | Enterobacterales | Enterobacteriaceae | Escherichia | Escherichia coli |
| Firmicutes | Clostridia | Eubacteriales | Lachnospiraceae | Roseburia | Roseburia inulinivorans |
| Firmicutes | Negativicutes | Acidaminococcales | Acidaminococcaceae | Phascolarctobacterium | Phascolarctobacterium succinatutens |
| Bacteroidetes | Bacteroidia | Bacteroidales | Bacteroidaceae | Bacteroides | Bacteroides stercoris |
| Firmicutes | Negativicutes | Selenomonadales | Selenomonadaceae | Mitsuokella | Mitsuokella multacida |
| Firmicutes | Clostridia | Eubacteriales | Eubacteriaceae | Eubacterium | Eubacterium sp. CAG:180 |
| Firmicutes | Negativicutes | Veillonellales | Veillonellaceae | Dialister | Dialister sp. CAG:486 |
| Firmicutes | Clostridia | Eubacteriales | Oscillospiraceae | Ruminococcus | Ruminococcus sp. CAG:177 |
| Bacteroidetes | Bacteroidia | Bacteroidales | Bacteroidaceae | Bacteroides | Bacteroides fragilis |
| Firmicutes | Negativicutes | Acidaminococcales | Acidaminococcaceae | Phascolarctobacterium | Phascolarctobacterium faecium |
| Bacteroidetes | Bacteroidia | Bacteroidales | Bacteroidaceae | Phocaeicola | Phocaeicola vulgatus |
| Bacteroidetes | Bacteroidia | Bacteroidales | Prevotellaceae | Prevotella | Prevotella sp. 885 |
| Bacteroidetes | Bacteroidia | Bacteroidales | Bacteroidaceae | Phocaeicola | Phocaeicola plebeius |
| Firmicutes | Clostridia | Eubacteriales | Oscillospiraceae | Faecalibacterium | Faecalibacterium prausnitzii |
| Bacteroidetes | Bacteroidia | Bacteroidales | Porphyromonadaceae | Porphyromonas | Porphyromonas gingivalis |
| Firmicutes | Bacilli | Lactobacillales | Streptococcaceae | Streptococcus | Streptococcus pneumoniae |
| Bacteroidetes | Bacteroidia | Bacteroidales | Prevotellaceae | Prevotella | Prevotella copri |

**Table S2**. The phylum and subsequent taxonomic levels of bacterium with LDA effect size (LEfSe) plots of pairwise analysis for immune non-responders and immune responders

| Phylum | Class | Order | Family | Genus | Species |
| --- | --- | --- | --- | --- | --- |
| Proteobacteria | Betaproteobacteria | Burkholderiales | Burkholderiaceae | Burkholderia | Burkholderia multivorans |
| Proteobacteria | Gammaproteobacteria | Vibrionales | Vibrionaceae | Vibrio | Vibrio vulnificus |
| Bacteroidetes | Bacteroidia | Bacteroidales | Bacteroidaceae | Phocaeicola | Phocaeicola plebeius |
| Proteobacteria | Gammaproteobacteria | Moraxellales | Moraxellaceae | Acinetobacter | Acinetobacter baumannii |
| Firmicutes | Clostridia | Eubacteriales | Eubacteriaceae | Eubacterium | Eubacterium sp. CAG:251 |
| Bacteroidetes | Bacteroidia | Bacteroidales | Prevotellaceae | Prevotella | Prevotella sp. CAG:5226 |
| Firmicutes | Negativicutes | Acidaminococcales | Acidaminococcaceae | Phascolarctobacterium | Phascolarctobacterium succinatutens |
| Firmicutes | Clostridia | Eubacteriales | Lachnospiraceae | Anaerobutyricum | Anaerobutyricum hallii |
| Bacteroidetes | Bacteroidia | Bacteroidales | Prevotellaceae | Prevotella | Prevotella sp. AM34-19LB |
| Bacteroidetes | Bacteroidia | Bacteroidales | Porphyromonadaceae | Porphyromonas | Porphyromonas gingivalis |

**Table S3**. Differential expression of inflammation-related proteins between treatment-naïve HIV-infected individuals and healthy controls

|  | TN | HC | *P* value | Adjusted_*P*val | |
| --- | --- | --- | --- | --- | --- |
| CXCL9 | 9.1127187 | 6.5630438 | 1.74E-14 | 1.29E-12 |  |
| TNFRSF9 | 6.8489407 | 5.3282558 | 2.39E-13 | 8.83E-12 |  |
| CXCL10 | 11.781277 | 9.2075713 | 5.56E-13 | 1.37E-11 |  |
| CXCL11 | 10.417431 | 7.7091817 | 1.32E-11 | 2.44E-10 |  |
| CD6 | 7.814088 | 6.2094958 | 1.23E-10 | 1.83E-09 |  |
| TNF | 4.5637283 | 3.2699873 | 1.35E-09 | 1.67E-08 |  |
| IL18 | 10.255172 | 9.036045 | 4.77E-09 | 5.04E-08 |  |
| CD5 | 7.1222883 | 6.1672275 | 1.71E-08 | 1.58E-07 |  |
| CCL19 | 10.077316 | 8.4184646 | 4.65E-08 | 3.82E-07 |  |
| CSF-1 | 10.271799 | 9.8403508 | 1.02E-07 | 7.52E-07 |  |
| TNFB | 5.0953997 | 4.2906804 | 2.37E-07 | 1.59E-06 |  |
| CD8A | 10.546936 | 8.0813904 | 8.56E-07 | 5.28E-06 |  |
| IFN-gamma | 7.635579 | 6.05221 | 2.88E-06 | 1.64E-05 |  |
| TRAIL | 7.4058173 | 6.8418038 | 4.50E-06 | 2.38E-05 |  |
| CCL23 | 10.888236 | 10.188993 | 4.96E-06 | 2.45E-05 |  |
| Flt3L | 9.2216817 | 8.6032138 | 5.61E-06 | 2.60E-05 |  |
| IL10 | 4.1908923 | 3.2726438 | 8.50E-06 | 3.70E-05 |  |
| IL-12B | 8.040853 | 6.4424996 | 9.11E-06 | 3.74E-05 |  |
| IL-18R1 | 8.7009113 | 8.0750992 | 1.33E-05 | 5.17E-05 |  |
| CCL20 | 8.7557967 | 7.4659658 | 1.92E-05 | 7.09E-05 |  |
| IL-15RA | 2.0917887 | 1.7153084 | 2.98E-05 | 0.0001049 |  |
| SCF | 9.11402 | 9.6478188 | 6.52E-05 | 0.0002192 |  |
| TRANCE | 5.0327563 | 4.2942221 | 9.96E-05 | 0.0003203 |  |
| CCL3 | 6.6400763 | 5.8311063 | 0.0001084 | 0.0003343 |  |
| CDCP1 | 3.0087037 | 2.1011795 | 0.0001514 | 0.0004482 |  |
| SLAMF1 | 3.0723276 | 2.7240487 | 0.0009343 | 0.0026593 |  |
| PD-L1 | 6.5774217 | 5.9289242 | 0.0052721 | 0.0144493 |  |
| MCP-1 | 11.815978 | 11.469149 | 0.0060084 | 0.0158793 |  |
| ADA | 6.7233347 | 6.2612138 | 0.0069258 | 0.0165764 |  |
| CX3CL1 | 3.974391 | 3.5583021 | 0.0070516 | 0.0165764 |  |
| IL-17C | 3.4151777 | 2.9757917 | 0.007149 | 0.0165764 |  |
| IL-10RB | 7.3362297 | 6.98108 | 0.0071682 | 0.0165764 |  |
| LAP TGF-beta-1 | 8.7432543 | 9.5656225 | 0.0086351 | 0.0193635 |  |
| uPA | 10.505825 | 10.14655 | 0.009069 | 0.0197385 |  |
| LIF-R | 3.553938 | 3.3795321 | 0.0100854 | 0.0213235 |  |
| VEGFA | 11.233007 | 10.778059 | 0.0181037 | 0.0372131 |  |
| TNFSF14 | 5.5745987 | 5.0259392 | 0.0214489 | 0.0428978 |  |

Data are presented as mean (normalized protein expression values, NPX).

Table S4 Differential expression of inflammation-related proteins between immune responders and healthy controls

|  | INR | HC | *P* value | Adjusted_*P*val |
| --- | --- | --- | --- | --- |
| LAP TGF-beta-1 | 8.0674417 | 9.5656225 | 5.90E-06 | 0.0004369 |
| CXCL11 | 8.972044 | 7.7091817 | 2.08E-05 | 0.0006238 |
| CCL25 | 6.7821157 | 5.9799604 | 2.53E-05 | 0.0006238 |
| CD8A | 9.8688917 | 8.0813904 | 7.44E-05 | 0.0013762 |
| CXCL10 | 9.9308717 | 9.2075713 | 9.38E-05 | 0.001388 |
| CCL11 | 8.4339497 | 7.914035 | 0.0002403 | 0.002964 |
| TRANCE | 3.5739543 | 4.2942221 | 0.0002891 | 0.0030564 |
| TNF | 3.7384667 | 3.2699873 | 0.0016661 | 0.0143467 |
| CCL20 | 8.4874403 | 7.4659658 | 0.0017449 | 0.0143467 |
| CXCL9 | 7.2438993 | 6.5630438 | 0.0030061 | 0.0222452 |
| IL18 | 9.630165 | 9.036045 | 0.0039627 | 0.026658 |
| MMP-10 | 8.4985047 | 7.178805 | 0.0062982 | 0.0370466 |
| TNFRSF9 | 5.660242 | 5.3282558 | 0.0065082 | 0.0370466 |
| MMP-1 | 14.253647 | 13.039793 | 0.0071693 | 0.0378948 |

Data are presented as mean (normalized protein expression values, NPX).

**Table S5**. Differential expression of inflammation-related proteins between immune non-responders and healthy controls

|  | IR | HC | *P* value | Adjusted_*P*val |
| --- | --- | --- | --- | --- |
| CXCL11 | 8.8176824 | 7.7091817 | 0.0002607 | 0.0192937 |
| CXCL10 | 9.8007831 | 9.2075713 | 0.0009787 | 0.0362115 |
| CCL25 | 6.5889903 | 5.9799604 | 0.0017644 | 0.043522 |

Data are presented as mean (normalized protein expression values, NPX).

**Table S6**. Differential expression of inflammation-related proteins between immune non-responders and immune responders

|  | INR | IR | *P* value | Adjusted_*P*val |
| --- | --- | --- | --- | --- |
| CD8A | 9.868892 | 8.435332 | 0.000159 | 0.011758 |
| LAP TGF-beta-1 | 8.067442 | 8.792037 | 0.000766 | 0.028346 |

Data are presented as mean (normalized protein expression values, NPX).
